# Celebrities’ impact on health-related knowledge, attitudes, behaviors, and status outcomes: protocol for a systematic review, meta-analysis, and meta-regression analysis

**DOI:** 10.1186/s13643-016-0395-1

**Published:** 2017-01-21

**Authors:** Steven J. Hoffman, Yasmeen Mansoor, Navneet Natt, Lathika Sritharan, Julia Belluz, Timothy Caulfield, Yoni Freedhoff, John N. Lavis, Arya M. Sharma

**Affiliations:** 1 Global Strategy Lab, Centre for Health Law, Policy and Ethics, Faculty of Law, University of Ottawa, Ottawa, Canada; 2 Department of Health Evidence and Impact and McMaster Health Forum, McMaster University, Hamilton, Canada; 3 Department of Global Health and Population, Harvard T.H. Chan School of Public Health, Boston, MA USA; 4 Faculty of Medicine, University of British Columbia, Vancouver, Canada; 5 Faculty of Medicine, University of Toronto, Toronto, Canada; 6 Vox Media, Washington, DC USA; 7 Health Law Institute, University of Alberta, Edmonton, Canada; 8 Bariatric Medical Institute, Ottawa, Canada; 9 Department of Family Medicine, Faculty of Medicine, University of Ottawa, Ottawa, Canada; 10 Centre for Health Economics and Policy Analysis and Department of Political Science, McMaster University, Hamilton, Canada; 11 Canadian Obesity Network and Faculty of Medicine, University of Alberta, Edmonton, Canada

**Keywords:** Famous persons, Public health, Preventive medicine, Health policy, Attitude to health, Health behavior

## Abstract

**Background:**

Celebrities are highly influential people whose actions and decisions are watched and often emulated by wide audiences. Many celebrities have used their prominent social standing to offer medical advice or endorse health products, a trend that is expected to increase. However, the extent of the impact that celebrities have in shaping the public’s health-related knowledge, attitudes, behaviors, and status is unclear. This systematic review seeks to answer the following questions: (1) Which health-related outcomes are influenced by celebrities? (2) How large of an impact do celebrities actually have on these health-related outcomes? (3) Under what circumstances do celebrities produce either beneficial or harmful impacts?

**Methods:**

Ten databases were searched, including MEDLINE, EMBASE, PsycINFO, PubMed, CINAHL, Communication Complete, Sociological Abstracts, Social Sciences Citation Index, Journals @ Scholars Portal, and ProQuest Dissertations & Theses A&I. Two reviewers conducted title and abstract screening and full-text screening to identify primary studies that employed empirical methods (either quantitative or qualitative) to examine celebrities’ impact on health-related knowledge, attitudes, behaviors, or status outcomes.

**Discussion:**

The results of this review will contribute to our understanding of celebrity influences and how to design positive evidence-based celebrity health promotion activities. In addition, these findings can help inform the development of media reporting guidelines pertaining to celebrity health news and provide guidance to public health authorities on whether and how to respond to or work with celebrities.

**Systematic review registration:**

PROSPERO CRD42015019268

**Electronic supplementary material:**

The online version of this article (doi:10.1186/s13643-016-0395-1) contains supplementary material, which is available to authorized users.

## Background

Celebrities can have a tremendous influence on the knowledge we retain, the attitudes we adopt, and the decisions we make, including those that affect our health [1–4]. A previous systematic meta-narrative analysis identified 14 biological, psychological, and social mechanisms through which celebrities influence our health-related behaviors, illustrating how the mechanics of celebrity and popular culture is a serious public health issue (see Table 1 for the 14 mechanisms) [5, 6]. This previous analysis identified those 14 mechanisms by systematically reviewing and synthesizing relevant research from economics, marketing, neuroscience, psychology, and sociology. The review of economics literature showed that celebrities can catalyze herd behavior, and help distinguish endorsed items from competitors. Marketing studies explained that celebrities’ characteristics are transferred to endorsed products which lends credibility to these products. These findings were supported by emerging neuroscience research which showed that brain regions involved in making positive associations are activated by seeing or hearing celebrity endorsements. The review of psychology literature showed that people are conditioned to react positively to celebrity advice and that are subconsciously pushed to follow it to avoid cognitive dissonance and to become more like those celebrities they admire. Finally, the sociology literature explained how the spread of celebrity advice through social networks increases its influence and that people follow this advice to acquire celebrities’ social capital [5, 6].

**Table 1:** Fourteen mechanisms explaining celebrity influence

Discipline	Mechanism	Description
Economics	1) Signals	Celebrity endorsements act as markers that differentiate endorsed items from competitors.
	2) Herd behavior	Celebrities activate people’s natural tendency to make decisions based on how others have acted in similar situations.
Marketing	3) Meaning transfer	People consume items to acquire the endorsing celebrities’ traits, which have become associated with the product.
	4) Source credibility	Celebrities share personal experiences and success stories associated with the endorsed item to be perceived as credible sources of health information.
	5) Halo effect	The specific success of celebrities is generalized to all their traits, biasing people to view them as credible medical advisors.
Neuroscience	6) Neural mechanisms of meaning transfer	Celebrity advertisements activate a brain region involved in forming positive associations, indicating the transfer of positive memories associated with the celebrity to the endorsed item.
	7) Neuropsychology of credibility	Endorsements from celebrities activate brain regions associated with trustful behavior and memory formation, thereby improving attitudes toward and recognition of the endorsed item.
Psychology	8) Classical conditioning	The positive responses people have toward celebrities come to be independently generated by endorsed items.
	9) Self-conception	People follow advice from celebrities who match how they perceive (or want to perceive) themselves.
	10) Cognitive dissonance	People unconsciously rationalize following celebrity medical advice to reduce the psychological discomfort that may otherwise result from holding incompatible views.
	11) Attachment	People, especially those with low self-esteem, form attachments to celebrities who make them feel independent in their actions, supported by others, and competent in their activities.
Sociology	12) Social networks	Celebrity advice reaches large masses by spreading through systems of people linked through personal connections.
	13) Commodification and social capital	People follow celebrity medical advice to gain social status and shape their social identities.
	14) Social constructivism	Celebrity medical advice may alter how people perceive health information and how it is produced in the first place.

Reproduced from Hoffman SJ, Tan C. Biological, psychological and social processes that explain celebrities’ influence on patients’ health-related behaviors. Archives of Public Health. 2015:73(3). doi:10.1186/2049-3258-73-3

Yet, despite this existing evidence about *why* people trust celebrities with their health, there is less evidence—and, to the best of our knowledge, no synthesized evidence—measuring the *magnitude* of this influence and the *conditions* that mediate it across different contexts.

Addressing this evidence gap is vitally important. On the one hand, celebrities may serve as an untapped resource for public health promotion efforts, where their influence could bring about positive changes in public opinion and health-related behaviors. This positive celebrity health effect was witnessed as early as the 1990s, after basketball player Earvin “Magic” Johnson announced that he was HIV-positive [7]. In the weeks following this disclosure, the US Centers for Disease Control and Prevention’s National AIDS Hotline reported over 28,000 calls from people expressing an increased concern about HIV/AIDS and seeking AIDS-related information [8].

Celebrity advocacy can also lead to the adoption of certain health prevention behaviors, as seen more recently with Angelina Jolie’s public announcement of her double mastectomy. Months later, studies recorded an increase in the number of high-risk patient screenings for the impugned *BRCA1* gene [9]. These studies suggest that celebrities can serve as agents of positive social change, erasing stigma associated with disease and prompting information-seeking and preventative behaviors. However, at the same time, the power of celebrities to sway public opinion can equally be a cause for concern [10–12]. Some authors criticize celebrities for presenting biased health information that evokes irrational fear and persuades audiences to behave in a certain way, rather than educating patients [11]. For example, Sabel and Sin discovered that many of the women who consulted a surgeon for an elective bilateral mastectomy following Jolie’s announcement were unsuitable candidates for the procedure following a thorough evaluation of their genome and family history [12]. Therefore, it may be important to combine celebrity advocacy with expert-led patient education in order to provide complete and accurate information about health issues and appropriately guide patient behavior [10–12].

Celebrities may also negatively affect the public’s health [13–15]. TV celebrity Jenny McCarthy’s anti-vaccine movement, for example, has captured significant public attention and roused concerns about vaccine safety [16–18]. Such celebrity advocacy can be counterproductive to the efforts of public health organizations that invest substantial resources in promoting the life-saving benefits of immunization [19]. With social media, it is now easier than ever for celebrities, journalists, and amateur bloggers to communicate directly with the public to influence their knowledge, attitudes, and/or behaviors; as technology further develops, this ability of non-experts to reach the masses will only increase and become more important to understand [20–22]. For example, in 2012, pop and R&B sensation Beyoncé signed a $50 million advertising contract with Pepsi—news that made national headlines [23]. This is not the first instance where famous musicians have been found to advertise unhealthy food items to the public through media outlets. A descriptive study by Bragg et al. found that 81% of foods endorsed by musicians featured in the Teen Choice Awards were of poor nutritional quality [24]. Given that youth and adolescents are a primary target of these promotional advertisements and moreover a significant at-risk group for obesity, these findings suggest inherent value in establishing guidelines that limit child exposure to celebrity food advertisements [13, 24]. As such, there is a potential opportunity for public health authorities to partner with celebrities, provided we better understand the impact of celebrity involvement and the conditions that determine this impact.

This planned review will compile empirical research evidence that evaluates the impact of celebrity health activities and the conditions that influence the direction and magnitude of that impact. To the extent possible, we will use a meta-analytic approach to synthesize the results from previous studies. Qualitative research will also comprise a critical component of our analysis, as we strive to better understand the health-related attitudes, behaviors, and preferences that are pervasive in society, their sociocultural underpinnings, and how celebrities may influence these beliefs. Ultimately, this review will use both quantitative and qualitative evidence to answer the following questions:

1. Which health-related outcomes are influenced by celebrities? We will focus on identifying the impact of celebrities’ advice on various health-related knowledge, attitudes, behaviors, and status outcomes. Health-related outcomes of interest will include a series of short-term, intermediate, and long-term outcomes, and are illustrated in the logic model (see Fig. 1).

**Fig. 1:**
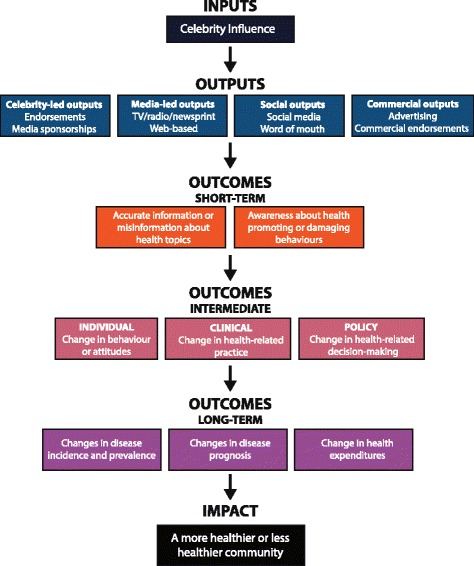
Logic model of the systematic review. This logic model illustrates the rationale and the interaction among the health-related outcome measures that will be assessed by this review
2. How large of an impact do celebrities actually have on these health-related outcomes? We aim to uncover both the directionality and the magnitude of celebrity impact on health by conducting a meta-analysis of quantitative studies.
3. Under what circumstances do celebrities produce either beneficial or harmful impacts? We will identify underlying and/or contextual factors that may also influence the impact of celebrities by conducting subgroup analyses of factors (i.e., demographical impact and location) as well as meta-regression analysis. Thematic synthesis of qualitative research will also shed light on individual factors that may render certain groups of individuals more susceptible to celebrity influence.

Currently, there only exists a fragmented collection of primary studies evaluating celebrities’ impact on health. Topics investigated by these primary studies include body image, cancer screening, smoking, and suicide. In addition, outcomes are evaluated across various populations and environments. Through the planned systematic review and meta-analysis, we will determine the extent to which the health effects of celebrity activities are consistent across the range of outcomes, populations, environments, and interventions. In order to effectively analyze such a heterogeneous pool of data and offer meaningful conclusions, studies will be categorized by common themes and outcomes. By encompassing a wide range of quantitative and qualitative evidence in our review, we aim to produce meaningful effect sizes to enhance our understanding of the present celebrity-health phenomenon and guide policymakers in facilitating significant and positive public health initiatives.

Overall, the results of this review should be helpful in understanding when to worry about negative celebrity influences and designing positive evidence-based celebrity health promotion activities. Findings could also help inform the development of media reporting guidelines pertaining to celebrity health news and provide guidance to public health authorities on whether and how to respond to or work with celebrities.

## Methods

### Study registration

We will conduct this systematic review, meta-analysis, and meta-regression analysis adhering to the following protocol and will report any changes to the protocol that arise as we proceed. The methods and design of this systematic review protocol are in accordance with the Preferred Reporting Items for Systematic Reviews and Meta-Analyses (PRISMA-P), available as an additional file to this protocol (see Additional file 1). This protocol is registered with PROSPERO (CRD42015019268).

### Types of study designs

Both quantitative and qualitative studies will be included in this systematic review to identify all empirical research evidence pertaining to the impact of celebrities on health-related outcomes.

All quantitative impact evaluations, including experiments (e.g., randomized controlled trials), quasi-experiments (e.g., interrupted time-series analyses), and observational designs (e.g., pooled time-series and cross-sectional analyses) will be included. Based on the results of our pilot search, we anticipate that most studies encountered will likely be observational in nature (see Additional file 2 for pilot search strategy). Within observational evaluations, both ecological studies (i.e., comparisons of groups rather than individuals) and individual-level studies (e.g., surveys of opinions) will be assessed. It is of value to include both ecological and individual-level studies since ecological studies alone face limitations with respect to identifying causal effects, whereas individual-level studies have an increased risk of reporting bias [25].

Qualitative studies will complement the knowledge gained from quantitative analyses, especially in providing rich insights into the contexts in which celebrities affect health-related outcomes. Qualitative research evidence will specifically allow us to better understand the meaning of the quantitative data, explore how individuals or groups may perceive certain celebrity interventions, and better understand the biological, psychological, and social mechanisms underlying the factors that mediate any influence.

### Types of participants

We will only include studies that evaluate the impact of celebrity health activities on individuals or groups of individuals. Studies that evaluate the impact of celebrities on corporations, governments, organizations, or similar entities will not be considered.

### Types of settings

Studies from all settings and countries will be considered, which is appropriate given that the influence of celebrities manifests itself in different places and across geography and culture.

### Types of interventions

Interventions include any health-related campaigns, news events, programs, or statements that primarily revolve around a celebrity or several celebrities—whether or not the celebrities intended to cause a health effect. Existing literature defines celebrity status in a number of ways, with a recent emphasis on celebrity as a type of capital that results from accumulated media visibility and recognizability [26, 27]. We aim to be liberal in our understanding of who are “celebrities,” with the recognition that various cultures may endow celebrity status in different ways and this may or may not be linked to media presence. Therefore, we chose to define celebrities as well-known and highly visible individuals in society, including but not limited to athletes, entertainment stars, media personalities, politicians, religious leaders, and socialites. Interventions will be included regardless of whether or not a celebrity played an active first-hand role in influencing a health-related outcome. In some cases, a celebrity may personally advocate for health-related behaviors; in other cases, a celebrity may be indirectly involved, such as a newspaper reporting on a particular health condition with which a celebrity was recently diagnosed. The goal is to broadly evaluate the impact of celebrities on health-related outcomes rather than only purposefully designed celebrity interventions that specifically aim to affect health-related outcomes.

Randomized controlled interventions are not necessary for a study to be included in this review. Examples of possible control interventions include, but are not limited to, comparator groups who were not exposed to the celebrity health activity. Studies without a control group will still be included in the review for the purposes of qualitative synthesis; however, only studies that assess the intervention in comparison to a control group will be included in the meta-analysis.

### Type of outcomes

The outcomes of interest in this review can be divided into short-, medium-, and long-term effects of celebrity intervention on health-related outcomes. In the short-term, celebrities may alter the public’s health-related knowledge, such as by changing the public’s understanding of a certain disease’s etiology, risk, diagnosis, and treatment. In the medium-term, celebrities may change the health-related attitudes and/or behaviors of the public. The Health Belief Model proposed that individuals’ perceptions of susceptibility, severity, benefits, and barriers related to health-related issues are precursors for their “readiness to act” and perform various health-related behaviors [28]. As such, we defined “health-related attitudes” to encompass this range of perceptions that help to shape individuals’ intentions to perform health-related behaviors. For instance, fear of needles may be viewed as a health-related attitude that will influence whether an individual seeks vaccination. A health behavior was originally defined by Kebl and Cobb as any activity undertaken by a person who believes himself to be healthy for the purpose of preventing disease or detecting disease in an asymptomatic stage [29]. For the purposes of this review, we are interested in examining both positive and negative celebrity influences as it applies to all individuals, both healthy and unhealthy, and thus defined a “health-related behavior” as any action that may promote or diminish one’s health status, be that physical, mental, or emotional well-being. For example, the decision of a high-risk patient to be screened for cancer would be a positive health behavior that can potentially benefit long-term health status, whereas declining treatment contrary to medical advice would be a negative health behavior that can lead to diminished health status. Finally, in the long-term, health status outcomes such as the incidence and prognosis of a preventable disease will be considered. These long-term health status outcomes are this review’s primary outcomes of interest; however, the secondary short- and medium-term outcomes serve as helpful secondary surrogate measures in the meta-analysis and potential mediating factors for study in the meta-regression analysis. Collecting evidence of celebrity influence on a wide range of primary and secondary outcomes from short- to long-term will allow for a comprehensive assessment of celebrity influence on health. The logic model illustrates this rationale and the interaction between these outcome measures in determining short-, medium-, and long-term health-related outcomes (see Fig. 1).

### Search strategy

We constructed a generic search strategy and adapted it for the MEDLINE database in consultation with health sciences and social sciences information specialists at McMaster University (see Table 2). With the goal of conducting the most comprehensive review possible, we aimed to maximize the sensitivity of our search by encompassing a broad range of potential celebrity interventions and health-related outcome measures in the search terms. We maintained specificity by taking a conservative approach to defining the inclusion and exclusion criteria (see “Selection of studies” section below).

**Table 2:** Generic search strategy and adapted search strategy for MEDLINE yielding 5157 records

Generic search strategy	(celebrit* or ((professional or elit* or famous or public or renown* or well-known or acclaim* or eminent or prominent or illustrious or recogniz* or reput* or influential or wealth* or power*) adj1 (person* or people or figure* or leader or athlete* or player or bodybuilder or sport* or basketball or football or hockey or baseball or soccer or Olympian or singer* or songwriter* or musician* or band or group or rapper* or artist* or actor* or actress or star or Hollywood or Bollywood or Nollywood or dancer or writer or author or comedian or performer or model* or supermodel* or chef or philanthropist or politic* or president or minister or king or queen or prince* or monarch))) AND (health or wellness or wellbeing or aging or longevity or disorder* or disease* or cancer or epidemic or pandemic or disability or impair* or ill* or sick* or ailment or malady or syndrome* or infection* or mortality or morbidity or death or dead or injur* or accident* or pain or incident*) AND (communicat* or promot* or endors* or advert* or convinc* or market* or persua* or dissua* or sell* or sale* or publici* or awareness or campaign or ‘media coverage’ or announc* or message* or disclos* or advoca* or advis* or advice or counsel* or educat* or instruct* or teach* or inform* or misinform* or prevent* or learn* or behavior?r* or act* or practice* or habit* or lifestyle* or regime or choice* or decision* or prefer* or attitude* or know* or belief* or perception* or view* or react* or response* or respond or stigma* or understand* or opinion* or litera* or illitera* or misunderstand* or misconcept* or misconstruct* or disbelief) AND (quantitative or qualitative or empirical or data or statistic* or evidence or stud* or “multi?method*” or survey* or interview or “self report*” or poll* or sampl* or experiment* or measure* or analyz* or analys* or ‘focus group*’ or question* or query or queri* or observation* or “field stud*” or phenomenolog* or phenomenograph* or ethnolog* or ethnograph* or “action research” or “grounded theory” or “case stud*”) AND (effect* or affect* or impact* or differ* or compliance or comply or adher* or implement* or influenc* or chang* or measure* or constrain* or screen* or control* or deter* or reduc* or increase* or decreas* or inflat* or vary or variation* or varie*)
MEDLINE search strategy (yielding 5157 records)	1. celebrit*.ti,ab. 2. ((professional or elit* or famous or public or renown* or well-known or acclaim* or eminent or prominent or illustrious or recogniz* or reput* or influential or wealth* or power*) adj1 (person* or people or figure* or leader or athlete* or player or bodybuilder or sport* or basketball or football or hockey or baseball or soccer or Olympian or singer* or songwriter* or musician* or band or group or rapper* or artist* or actor* or actress or star or Hollywood or Bollywood or Nollywood or dancer or writer or author or comedian or performer or model* or supermodel* or chef or philanthropist or politic* or president or minister or king or queen or prince* or monarch)).ti,ab. 3. exp Famous Persons/ 4. 1 or 2 or 3 5. exp Health Promotion/ 6. exp preventive health services/or exp health education/ 7. exp attitude to health/or exp health knowledge, attitudes, practice/ 8. exp Health Communication/ 9. exp health behavior/or exp information seeking behavior/ 10. exp information dissemination/or exp information literacy/or exp health literacy/ 11. exp Public Opinion/ 12. (communicat* or promot* or endors* or advert* or convinc* or market* or persua* or dissua* or sell* or sale* or publici* or awareness or campaign or “media coverage” or announc* or message* or disclos* or advoca* or advis* or advice or counsel* or educat* or instruct* or teach* or inform* or misinform* or prevent* or learn* or behavior?r* or act* or practice* or habit* or lifestyle* or regime or choice* or decision* or prefer* or attitude* or know* or belief* or perception* or view* or react* or response or respond* or stigma* or understand* or opinion* or litera* or illitera* or misunderstand* or misconcept* or misconstruct* or disbelief).tw. 13. 5 or 6 or 7 or 8 or 9 or 10 or 11 or 12 14. (health or wellness or wellbeing or aging or longevity or disorder* or disease* or cancer or epidemic or pandemic or disability or impair* or ill* or sick* or ailment or malady or syndrome* or infection* or mortality or morbidity or death or dead or injur* or accident* or pain or incident*).tw. 15. exp Public Health/ 16. 14 or 15 17. (quantitative or qualitative or empirical or data or statistic* or evidence or survey or stud* or interview or ‘self report*’ or poll* or experiment* or measure* or analyz* or analys* or ‘focus group*’ or question* or query or queri* or observation* or ‘field stud*’ or phenomenolog* or phenomenograph* or ethnolog* or ethnograph* or ‘action research’ or ‘grounded theory’ or ‘case stud*’ or ‘multi?method*’).mp. 18. exp empirical research/or exp qualitative research/ 19. exp health surveys/or exp interviews as topic/or exp focus groups/or exp questionnaires/or exp self report/or exp sampling studies/or exp sample size/or exp observation/ 20. 17 or 18 or 19 21. (effect* or affect* or impact* or differ* or compliance or comply or adher* or implement* or influenc* or chang* or measure* or constrain* or screen* or control* or deter* or reduc* or increase* or decreas* or inflat* or vary or variation* or varie*).tw. 22. 4 and 13 and 16 and 20 and 21

We adapted the generic search strategy for the following ten electronic databases (see Additional file 3 for all adapted search strategies): CINAHL (1982 to July 2014); Communication Complete (1915 to July 2014); EMBASE (1947 to July 2014); Journals @ Scholars Portal (no date restrictions); OVID MEDLINE In-Process & Other Non-Indexed Citations (1946 to July 2014); Proquest Dissertations & Theses A&I electronic database (all dates); PsycINFO (1806 to July 2014); PubMed (1966 to July 2014); Social Sciences Citation Index (1976 to July 2014); and Sociological Abstracts (1952 to July 2014). We searched a combination of health and social sciences databases and additionally looked for gray literature. All searches were run on July 31, 2014. There were no language restrictions and the databases were searched from their earliest date of inception.

### Selection of studies

Two members of the research team (YM/NN) independently screened the title and abstract of each study for inclusion based on a pre-established eligibility screening form (Additional file 4). This eligibility criteria form was used to screen for both quantitative and qualitative studies and, moreover, helped to categorize which studies belonged to which study design. Articles were selected during the title/abstract screening if reviewers answered “yes” to all three questions:

- *Independent variable*: Does the study explore a potential association with celebrities as the intervention?
- *Dependent variable*: Does the study explore a potential association with outcomes associated with health-related knowledge, attitudes, behaviors, and/or status? (Note that health-related behaviors encompass a wide range of actions that can either promote or threaten one’s health)
- *Method*: Is the study an empirical evaluation? (Either quantitative or qualitative)

**Fig. 2:**
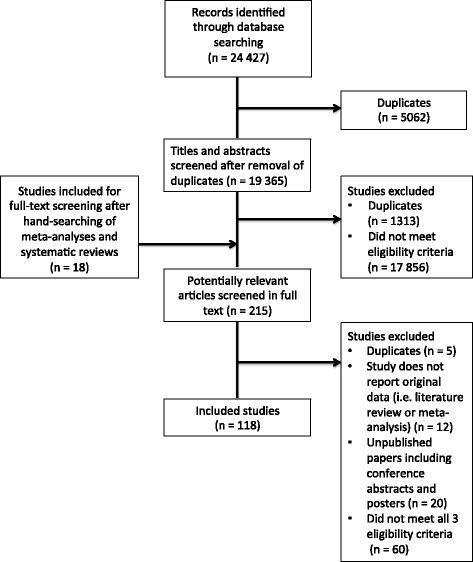
PRISMA flowchart of the systematic review. This flowchart outlines the process of database searches, hand searches, title and abstract screening, and full-text screening, and lists the number of studies included and excluded with reasons

Disagreements were resolved through discussion between the two reviewers. If there had been a case in which a consensus could not be reached, the principal investigator (SJH) would have been consulted, although that was never necessary. Studies that were eligible for inclusion based on title and abstract screening were then reviewed in a full-text screening exercise. The same inclusion criteria were used during full-text screening as the title/abstract screening but with two adjustments: first, the full-text screening required a precise examination of whether celebrity interventions were being evaluated and if a health-related knowledge, attitude, behavior, or status outcome was being measured; and second, we narrowed our third eligibility criteria to only include empirical studies that had been *peer-reviewed*. Doing so enhanced the specificity of our search, allowing us to exclude studies that were likely to be less robust and credible. Again, the two independent reviewers used an eligibility screening form to determine which studies met the full-text inclusion criteria (see Additional file 5 for the full-text screening form). Disagreements were resolved through discussion; if consensus could not be reached, the principal investigator would have been consulted, although that was never necessary. Figure 2 visualizes the first part of a PRISMA flowchart showing the number of articles included and excluded at each stage of screening.

### Data extraction from quantitative studies

Given the likely variability among studies and the challenges we anticipate in developing a standardized form that can be applied to all included studies, three stages of data extraction will be conducted. In the first stage, general study information will be gathered including details about participant demographics, interventions, and methods employed (see Additional file 6 for a draft form for stage one of data extraction). This will help make data extraction more efficient by grouping together studies with similar study designs. The next phase of data extraction will focus specifically on defining outcome variables. This information will be used to further group studies by outcomes, thus facilitating the development of a third data extraction form specifically tailored to each subgroup of studies. The third data extraction form will focus on collecting precise quantitative data and key findings specific to the outcome measures reported in the second data extraction form. As there will be a large number of studies in this review, data extraction will be completed by two pairs of extractors (four reviewers total), with each pair randomly assigned half of the studies.

The three data extraction forms will be developed through a series of calibration exercises involving the investigators, data extractors, and a statistician. Specifically, each extractor will independently extract data from three purposively selected studies with diverse methodologies and celebrity activities. After data extraction is performed, discrepancies will be discussed until full agreement is reached among the extractors. We will also ask each extractor for feedback to modify the data extraction forms such that they are user-friendly and applicable to the wide range of studies that are included. These calibration exercises will be repeated until all four reviewers agree on a reliable set of data extraction forms and achieve near-perfect consistency in the extraction process. After finalizing the standardized data extraction forms, they will be implemented in Distiller SR online software to maximize efficiency of the data extraction process. Similar to the calibration exercises, disagreements will be resolved by discussion between reviewers to achieve consensus and consultation with the principal investigator if necessary.

### Data extraction from qualitative studies

Two extractors will independently describe the main findings from the included qualitative studies using a standardized form implemented in Distiller SR. Extractors will record specific text fragments from the studies wherever possible and appropriate. In line with our third research question, we will also extract information on any factors identified as mediators of celebrities’ impact on health-related outcomes and make note of any themes or trends that are uncovered. Overall themes across all included qualitative studies will be allowed to emerge naturally from the data in keeping with thematic synthesis methodology; they will not be pre-identified by a priori hypotheses [30, 31]. Any discrepancies about the details and conclusions from qualitative studies will be addressed through discussion between reviewers, consulting the principal investigator if consensus is not possible.

### Assessment of risk of bias in included studies

Studies that met the inclusion criteria will be assessed for bias using either the Cochrane Risk of Bias Tool for Randomized Trials or the Cochrane Risk of Bias in Non-Randomized Studies for Interventions (ROBINS-I) tool, depending on study design [32, 33]. Although the kinds of biases are broadly equivalent for randomized and non-randomized studies—namely selection, confounding, group equivalence, spill-overs, and reporting biases—there are important differences in their operationalization. Raters will resolve disagreements by discussion and through consultation with the principal investigator if necessary. Qualitative studies will be assessed for quality using the Critical Appraisal Skills Programme (CASP) approach, given its widely cited use in qualitative reviews and recommendation from the Cochrane Qualitative Research Methods Group. We will explore reporting biases such as publication bias by using funnel plots and assessing the asymmetry both visually and using the Egger test [34]. In the presence of visual asymmetry, exploratory analyses will be performed.

### Data analysis

Studies will first be categorized by outcome domain (short-term, medium-term, and long-term health-related outcomes). Within each outcome domain, three types of analysis will be conducted, resulting in at least three sets of results: 1) summaries of studies demonstrating that celebrities have positive, negative, mixed, or no impacts on the health-related outcome; 2) synthesized quantitative measurements of the direction and magnitude of any identified impact that celebrities have on the health-related outcome; and 3) evidence identifying the comparative influence of different factors, circumstances, or conditions that mediate whether celebrities have positive or negative effects on the health-related outcome. A meta-analysis will facilitate all three lines of inquiry, while the subgroup analyses, meta-regression analysis, and the qualitative review will contribute to the third line of inquiry.

#### Meta-analysis

In preliminary searches of health sciences and social sciences databases, it was found that studies evaluating celebrities’ impact on health were very heterogeneous. Studies investigated a variety of outcomes, ranging from HIV knowledge to opinions on different foods and to cancer screening behaviors. In addition, these outcomes were evaluated across various populations, environments, and interventions. Through the meta-analysis, we will determine the extent to which the effects of celebrity influence on health are consistent across this range of outcomes, populations, environments, and interventions. For questions in which it is plausible, we will conduct meta-analyses.

Our aim is to be liberal in this judgment. We will pool results from a relatively broad range of studies of different outcomes, populations, environments, and interventions. Having done that, we can examine the variability in results to determine the extent to which the data supports the assumption regarding similar effects across outcomes, populations, environments, and interventions. We anticipate substantial variability and will explore this through pooling across studies that share similar outcomes and conducting subgroup analyses and/or meta-regression analysis (see below). This approach will allow us to estimate a broad summary effect of celebrities on health and also estimate more specific celebrity effects.

For dichotomous outcomes, the risk ratio or odds ratio and 95% confidence intervals will be used for pooling the data. For continuous outcomes, mean differences and 95% confidence intervals will be used for pooling outcomes reported on the same scales or measured in the same units. Data will be transformed to allow analyses with mean differences wherever possible. All formats of continuous outcome data will be extracted whether reported as post-intervention or change from baseline. We will consider using the *R* value, a correlation coefficient, for continuous variables when pooling some studies that report dichotomous outcomes and others that report continuous outcomes. If so, results from individual studies will be converted to an *R* value before the meta-analysis. The *R* value can be roughly interpreted as a small (*R* = 0.1), medium (*R* = 0.3), or large (*R* = 0.5) effect size. We plan to transform the pooled *R* to another statistic, such as an odds ratio, to aid in interpretation. For time-to-event data, the hazard ratio, which is usually estimated from a Cox proportional hazards model, will be pooled using the generic inverse variance method.

Since we know that the studies will measure outcomes of interest in different units, synthesized effect sizes of continuous outcomes will additionally be measured using the standardized mean difference (SMD) (also sometimes labeled the “effect size”). This will be calculated by dividing the difference in mean values by the pooled standard deviation of the two groups (i.e., intervention versus non-intervention), as outlined in the following equation:

$$ \mathrm{S}\mathrm{M}\mathrm{D}=\frac{\mathrm{Differenceinmeanoutcomebetweengroups}}{\mathrm{Standarddeviationofoutcomeamongparticipants}} $$

This measure will allow us to compare and pool across studies where outcomes are reported in different units. The SMD will be expressed as a ratio of means to facilitate meaningful interpretation of the effect size.

In interpreting the SMDs, a rule of thumb suggests that 0.2 standard deviation units represents a small effect, 0.5 a moderate effect, and 0.8 a large effect. In addition to this rule of thumb, to facilitate our interpretation, we will convert the SMDs to measures of effect typically used for binary outcomes. In principle, this approach assumes that data are normally distributed, allowing calculation of the probability that results are greater than equal to a particular threshold. These probabilities allow calculation of odds ratios and risk differences. There are a number of methods available to conduct the conversion from continuous to binary outcomes. We will use the approach described by Furukawa [35].

Effect sizes for non-continuous outcomes will be measured by combining risk ratios. Ninety-five percent confidence intervals will be calculated for all effect measures. We will be conservative in our methodology by employing a random-effects meta-analysis as it considers both within- and between-study heterogeneity. We will statistically examine the extent of heterogeneity using chi-square tests of heterogeneity and measure the extent of inconsistency with the *I* ^2^ measure.

#### Subgroup analyses

Several subgroup analyses are planned to quantitatively measure the impact of different kinds of celebrity activities depending on their source, message, and audience, assuming there are a sufficient number of studies with relevant data to allow for it. These include the following:

- *Source: celebrity type.* At least one existing study has shown that entertainment stars may be more influential than politicians in terms of influencing copycat suicide attempts [36]. This suggests the celebrity’s occupation may be an important mediating factor. Types of celebrities that will be compared include athletes, entertainment stars, media personalities, politicians, religious leaders, and socialites.
- *Message: communication channel*. As forms of media differ so greatly, so too may the impact of a celebrity health activity disseminated through different channels [37]. Media differ in their typical length of exposure, sensory effects, and tone. Audiovisual (television/film), print, radio, and social media will be compared.
- *Message: time period*. Are celebrities more influential on our health in today’s age of social media than ever before? We will group and compare studies that evaluated celebrity health activities during different time periods. At the very least, we plan to group studies conducted before and after 2004—the year Facebook launched.
- *Message: tone*. The impact of celebrities may differ based on whether it is disseminated in a positive of negative tone. Positive dissemination involves conveying a particular opinion or action in a favorable light, while negative dissemination warns the public against an opinion or action. For example, one study found that negative reporting of celebrity suicides was associated with 99% fewer copycat suicides [37]. As possible, studies will be dichotomized according to whether they evaluated positively and negatively framed celebrity activities to measure the potential influence of tone.
- *Message: condition/risk type*. Given people often have strong prior perceptions, the impact of celebrities may not only be due to their influence alone, but also the public’s existing views about particular conditions and risks. As data allows, celebrities’ impact will be assessed across several broad classes of conditions and risk factors, hopefully including cancer, mental illness, physical disability, sexually transmitted disease, vaccination, and consumption of harmful products like alcohol, narcotics, and tobacco.
- *Audience: demographics.* Certain populations may be more susceptible to celebrity influence than others, on the basis of age, culture, education, gender, or socioeconomic status [7]. Studies will be stratified by demographic factors, depending on the availability of demographic data in the included studies. Studies will also be stratified by World Bank regional groupings of countries to see whether the effect of celebrity influence differ across countries.

We will additionally conduct a subgroup analysis of the highest-quality studies to calculate a more conservative broad measure of celebrities’ impact on health-related outcomes.

#### Meta-regression

Meta-regression will be used to explore the reasons for different effect sizes across studies which will help us to quantify the specific influence of various factors that might determine celebrities’ impact. Depending on the number of studies available from which results can be analyzed in this way, we will endeavor to consider as many factors as possible from the list previously identified for the planned subgroup analyses (see above) as well as any others that might be relevant and feasible such as the study’s design. This analysis will be especially helpful for developing media reporting guidelines on how to cover celebrity health news and providing guidance to public health authorities on whether and how to respond to or work with celebrities.

#### Synthesis of qualitative results

Thematic synthesis will be used to synthesize the data from the qualitative studies. This approach combines free-coding, iterative categorization of text fragments, and reciprocal translational analysis from meta-ethnography with grounded theory’s inductive approach and constant comparison method [38, 39]. We will aim to synthesize the themes that emerge—particularly those on the factors and mediators of celebrity influence on health-related outcomes—into a conceptual framework that can inform both understanding and future inquiry. The conceptual framework will supplement the subgroup analyses and meta-regression analysis in order to make recommendations on how to minimize the harm and maximize the benefits that celebrity health activities may cause in a range of contexts.

## Discussion

Given that this is the first review of its kind, we anticipate several future challenges as we strive to evaluate both the extent and magnitude of celebrities’ impact on different health-related outcomes. Nevertheless, we have proactively undertaken several steps to overcome these anticipated challenges in order to conduct a rigorous systematic review.

Specifically, when defining our independent variable, we recognized that celebrities can include many different types of public figures, such that we adopted a broad definition that can be operationalized across varied cultures and countries. Additionally, we acknowledged that celebrity interventions could assume many different forms. Given these many considerations, we opted for a broad definition of celebrity interventions in order to ensure a more comprehensive review of the literature and retrieve as many relevant studies as possible. We took the same approach when assigning definitions for the dependent variables of interest, including what comprises health-related attitudes and behaviors. The use of broad definitions allowed us to conduct a highly sensitive literature search that yielded 19,365 records that were each reviewed.

One future obstacle that we foresee involves the analysis of both quantitative and qualitative studies in our analysis. In order to maximize the relevance of this study for policymakers in their decision-making processes, we feel it necessary to incorporate diverse forms of evidence. While quantitative studies provide a highly precise means to assess celebrity impact, we cannot discount the importance of ethnographic research and other qualitative findings since celebrity influence is a culturally based and sociologically rooted phenomenon. A significant challenge therefore lies in finding the most effective way to synthesize our qualitative and quantitative findings to answer the three research questions. We will address this issue by developing two independent strategies for data analysis—one for quantitative studies and the other for qualitative studies. As possible, quantitative evaluations will be subject to meta-analysis so as to pool different effect sizes and determine the magnitude of celebrity influence for different health-related outcomes. Meta-regression and subgroup analyses will offer additional insights as to which contextual factors either enhance or diminish the aforementioned celebrity effect. Qualitative studies will complement the knowledge gained from quantitative analyses and provide additional understanding of the factors affecting the celebrity-health phenomenon. For example, by analyzing the outcomes of focus groups and surveys, we can draw upon individual perspectives and community experiences to better understand the types of attitudes that exist toward various health topics, the factors that may shape these attitudes, and the exact motives driving certain health-related behaviors. Conducting two distinct methods of analysis will preserve the unique benefits offered by quantitative and qualitative methodologies, and will allow us to draw from different pools of findings to facilitate a more critical analysis of our research questions.

Finally, there are many considerations to take into account when ensuring our review has sufficient power. From our initial pilot search (Additional file 2), we uncovered a vast amount of literature examining celebrity impacts on different health topics such as smoking, body image, suicide, and cancer screening. In order to effectively analyze such a heterogeneous pool of data and provide meaningful conclusions, we must carefully categorize all included studies by themes and outcomes before performing any statistical tests. This has been addressed by conducting data extraction in three distinct stages, with the first two stages analyzing the study designs and outcome measures in order to group them before proceeding with the final phase of data extraction. While organizing the studies in this way is crucial to achieving a meaningful analysis, it may decrease the number of studies available for statistical testing with respect to each outcome. However, by encompassing a wide range of quantitative and qualitative evidence in our study, we aim to produce meaningful findings to enhance our understanding of the present celebrity-health phenomenon and guide policymakers in facilitating significant and positive public health initiatives.

In order for this review to be leveraged to have a meaningful impact on public health, our research team is committed to engaging in a series of knowledge translation activities. By disseminating our findings to a wide range of stakeholders in the public health arena, health systems can involve celebrities in their efforts to promote positive health messages through opportunistic moments while working in partnership to mitigate the potentially negative impact that celebrities might have. These knowledge translation activities will be specifically targeted toward the development of updated media reporting guidelines, partnerships with celebrities, and future public health interventions.

## Additional files

Additional file 1: Populated PRISMA-P (Preferred Reporting Items for Systematic Review and Meta-Analysis Protocols) checklist. Recommended items to include in a systematic review protocol. (DOCX 102 kb)
Additional file 2: Pilot search strategy. Reports the search phrases and the databases on which the pilot search was conducted. (DOCX 117 kb)
Additional file 3: Adapted search strategies for each database. (DOCX 147 kb)
Additional file 4: Title and abstract screening form. (DOCX 80 kb)
Additional file 5: Full-text screening form. (DOCX 81 kb)
Additional file 6: Data abstraction form —phase 1. The first data abstraction form that will be utilized with all studies in order to collect population, intervention, methodology, and preliminary outcome information. (DOCX 111 kb)

## Abbreviations

CINAHL: Cumulative Index to Nursing and Allied Health Literature
HIV/AIDS: Human immunodeficiency virus infection and acquired immune deficiency syndrome
SMD: Standardized mean difference
