# Supplementary material for: Celebrities’ impact on health-related knowledge, attitudes, behaviors, and status outcomes: protocol for a systematic review, meta-analysis, and meta-regression analysis
Source: Syst Rev. 2017 Jan 21;6:13. doi: 10.1186/s13643-016-0395-1 (PMC5251292; doi:10.1186/s13643-016-0395-1)
Supplement: Additional file 2: — Pilot search strategy. Reports the search phrases and the databases on which the pilot search was conducted. (DOCX 117 kb) [file 13643_2016_395_MOESM2_ESM.docx]

**Additional File 2**

Pilot Search Strategies

**May 21, 2014**

| Field | Database and Justification | Search Phrase | # Yielded | # Unique Potentially Relevant Titles |
| --- | --- | --- | --- | --- |
| General and Multidisciplinary | ProQuest Databases,  *Highly comprehensive database spanning a breadth of different topics* | Celebrit* AND health AND public  Limit to: conference papers & proceedings, dissertations & theses, government & official publications, scholarly journals, working papers  Some journals which popped up frequently and may be relevant to hand-search:  -British Medical Journal (5)  -Journal of Health Communication (5)  -Health Promotion International (4)  -Health Communication (5) | 310 | 93 (1 non-english) |
|  |  | Celebrit* AND (sport or athlete*) AND “public health” | 16 | 8 (all duplicates) |
|  | Excluded those reviews which examined reverse association (perceptions of celebrities by those dissatisfied with their own body) | Celebrity AND “body image” | 61 | 35 |
| May 23, 2014 | Proquest Database | Celebrit* AND “public health” AND (campaign or endors*)  Limit to: conference papers & proceedings, dissertations & theses, government & official publications, scholarly journals, working papers | 102 | 0 |
|  |  | Celebrit* AND “public health” AND (drug or “substance use”)  *Goal: to see if a specific public health outcome* | 33 | 0 |
| General and Multidisciplinary | EBSCOhost Database  (searched in order to further assess the types of papers out there before continuing on to more specific databases) | Celebrit* AND impact AND public AND health  1900-2015  Academic journals only  **started with a specific search strategy that could be expanded afterwords* | 28 | 2 |
|  |  | (Celebrit* or “public figure*” or “famous person*”) AND impact AND health  Limited to academic journals only | 6,422 | Too many irrelevant articles upon initial screening |
|  |  | Celebrit* AND campaign AND health AND (attitude* or behaviour*)  Limited to academic journals only | 708,759 | Many were not focussed on health |
|  | Social Sciences Abstracts  Due to the high volume of searches retrieved from EBSCOhost, decided to go one by one through individual databases, starting off with those which would examine celebrity influence from a social sciences point of view | Celebrit* AND public AND health | 28 | 0 |
|  |  | Celebrit* AND health | 48 | 4 |
|  |  | (Celebrit* or “famous person*” or “public figure*”) AND “health promotion” | 443 | 0 |
|  |  | (Celebrit* or “famous person*” or “public figure*”) AND impact AND (“health attitude*” or “health behavior*) | 443 | 0 |
| Social Sciences | ASSIA – Applied Social Sciences Index and Abstracts  *Covers both social sciences and health information from 650 different journals across 16 different countries; the previous social sciences database did not retrieve many health-related articles thus this seemed more useful* | (Celebrit* or “famous person*” or “public figure*”) AND “health” | 77 | 0 |
|  |  | (celebrit* OR "famous person*" OR "public figure*") AND health AND (campaign OR promot* OR endors* OR advoca* OR advertis*) | 23 | 0 |
| Social Sciences | Communication Abstracts  *The initial general search through ProQuest revealed a lot of articles that were published in communications journals. Felt it would be appropriate to further search this field* | (Celebrit* or “famous person*” or “public figure*”) AND “health” | 50 | 4 |
|  |  | (Celebrit* or “famous person*” or “public figure”) AND health AND (campaign OR endors* OR promot* OR advoca* OR advert*) | 23 | 0 |
|  | *decided to narrow search to more specific health impacts | (Celebrit* or “famous person*” or “public figure”) AND (nutrition OR diet OR food) | 15 | 0 |
|  |  | (Celebrit* or “famous person*” or “public figure”) AND “body image” | 0 | 0 |
|  | Communication and Mass Media Complete *More comprehensive than Communication Abstracts* | (Celebrit* or “famous person*” or “public figure*”) AND “health” | 105 | 4 |
| Grey Literature | Dissertations & Theses  *Thought this would be a good source of grey literature* | (Celebrit* or “famous person*” or “public figure*”) AND “health” | 1 | 0 |
| Health Sciences | PsycINFO  *Many of the articles previously retrieved from ProQuest cited PsycINFO* | (all(celebrit*) OR all("famous people") OR all("famous person*") OR all("public figure*")) AND all(health) AND (all(campaign) OR all(promot*) or all(advertis*) or all(endors*) or all(advoca*)) | 114 | 3 |
|  | CINAHL |  |  |  |
| May 26, 2014 | Google Scholar | Celebrity AND public health impact | 67,700 |  |
|  | Proquest  (Returned to ProQuest to see how variations in terminology affect specificity vs. sensitivity of search; based on the 143 articles that seemed relevant from preliminary title scan over the past few days) | Celebrit* AND health AND (impact OR effect* OR influenc*) | 301 |  |
|  |  | Celebrit* AND “public health” | 132 | 49 (previously included) |
|  |  | Celebrit* AND “public health” AND (impact OR effect* OR influenc*) | 67 | 34  (previously included) |
|  |  | Celebrit* AND health AND (campaign OR endors* OR advertis* OR advoca* OR testimon*) | 218 |  |
|  |  | Celebrit* AND “public health” AND (campaign OR endors* OR advertis* OR advoca* OR testimon*) | 68 |  |
