# Supplementary material for: Celebrities’ impact on health-related knowledge, attitudes, behaviors, and status outcomes: protocol for a systematic review, meta-analysis, and meta-regression analysis
Source: Syst Rev. 2017 Jan 21;6:13. doi: 10.1186/s13643-016-0395-1 (PMC5251292; doi:10.1186/s13643-016-0395-1)
Supplement: Additional file 3: — Adapted search strategies for each database. (DOCX 147 kb) [file 13643_2016_395_MOESM3_ESM.docx]

**Additional File 3**

Adapted Search Strategies

Medical Databases

Date of searches: 2014-07-31

**MEDLINE**

Total yield = 5157

1. celebrit*.ti,ab.

2. ((professional or elit* or famous or public or renown* or well-known or acclaim* or eminent or prominent or illustrious or recogniz* or reput* or influential or wealth* or power*) adj1 (person* or people or figure* or leader or athlete* or player or bodybuilder or sport* or basketball or football or hockey or baseball or soccer or Olympian or singer* or songwriter* or musician* or band or group or rapper* or artist* or actor* or actress or star or Hollywood or Bollywood or Nollywood or dancer or writer or author or comedian or performer or model* or supermodel* or chef or philanthropist or politic* or president or minister or king or queen or prince* or monarch)).ti,ab.

3. exp Famous Persons/

4. 1 or 2 or 3

5. exp Health Promotion/

6. exp preventive health services/ or exp health education/

7. exp attitude to health/ or exp health knowledge, attitudes, practice/

8. exp Health Communication/

9. exp health behavior/ or exp information seeking behavior/

10. exp information dissemination/ or exp information literacy/ or exp health literacy/

11. exp Public Opinion/

12. (communicat* or promot* or endors* or advert* or convinc* or market* or persua* or dissua* or sell* or sale* or publici* or awareness or campaign or "media coverage" or announc* or message* or disclos* or advoca* or advis* or advice or counsel* or educat* or instruct* or teach* or inform* or misinform* or prevent* or learn* or behavior?r* or act* or practice* or habit* or lifestyle* or regime or choice* or decision* or prefer* or attitude* or know* or belief* or perception* or view* or react* or response or respond* or stigma* or understand* or opinion* or litera* or illitera* or misunderstand* or misconcept* or misconstruct* or disbelief).tw.

13. 5 or 6 or 7 or 8 or 9 or 10 or 11 or 12

14. (health or wellness or wellbeing or aging or longevity or disorder* or disease* or cancer or epidemic or pandemic or disability or impair* or ill* or sick* or ailment or malady or syndrome* or infection* or mortality or morbidity or death or dead or injur* or accident* or pain or incident*).tw.

15. exp Public Health/

16. 14 or 15

17. (quantitative or qualitative or empirical or data or statistic* or evidence or survey or stud* or interview or 'self report*' or poll* or experiment* or measure* or analyz* or analys* or 'focus group*' or question* or query or queri* or observation* or 'field stud*' or phenomenolog* or phenomenograph* or ethnolog* or ethnograph* or 'action research' or 'grounded theory' or 'case stud*' or 'multi?method*').mp.

18. exp empirical research/ or exp qualitative research/

19. exp health surveys/ or exp interviews as topic/ or exp focus groups/ or exp questionnaires/ or exp self report/ or exp sampling studies/ or exp sample size/ or exp observation/

20. 17 or 18 or 19

21. (effect* or affect* or impact* or differ* or compliance or comply or adher* or implement* or influenc* or chang* or measure* or constrain* or screen* or control* or deter* or reduc* or increase* or decreas* or inflat* or vary or variation* or varie*).tw.

22. 4 and 13 and 16 and 20 and 21

**EMBASE**

Total yield = 4955

1. celebrit*.ti,ab.

2. ((professional or elit* or famous or public or renown* or well-known or acclaim* or eminent or prominent or illustrious or recogniz* or reput* or influential or wealth* or power*) adj1 (person* or people or figure* or leader or athlete* or player or bodybuilder or sport* or basketball or football or hockey or baseball or soccer or Olympian or singer* or songwriter* or musician* or band or group or rapper* or artist* or actor* or actress or star or Hollywood or Bollywood or Nollywood or dancer or writer or author or comedian or performer or model* or supermodel* or chef or philanthropist or politic* or president or minister or king or queen or prince* or monarch)).ti,ab.

3. exp public figure/

4. 1 or 2 or 3

5. exp health education/ or exp health literacy/ or exp health promotion/

6. exp medical information/

7. exp health behavior/ or exp health belief/

8. exp attitude to death/ or exp attitude to disability/ or exp attitude to health/ or exp attitude to illness/ or exp attitude to life/ or exp attitude to mental illness/ or exp attitude to aging/

9. exp public opinion/

10. exp information seeking/

11. exp information dissemination/ or exp information literacy/

12. exp preventive health service/ or exp preventive medicine/

13. (communicat* or promot* or endors* or advert* or convinc* or market* or persua* or dissua* or sell* or sale* or publici* or awareness or campaign or "media coverage" or announc* or message* or disclos* or advoca* or advis* or advice or counsel* or educat* or instruct* or teach* or inform* or misinform* or prevent* or learn* or behavior?r* or act* or practice* or habit* or lifestyle* or regime or choice* or decision* or prefer* or attitude* or know* or belief* or perception* or view* or react* or response or respond* or stigma* or understand* or opinion* or litera* or illitera* or misunderstand* or misconcept* or misconstruct* or disbelief).tw.

14. 5 or 6 or 7 or 8 or 9 or 10 or 11 or 12 or 13

15. (health or wellness or wellbeing or aging or longevity or disorder* or disease* or cancer or epidemic or pandemic or disability or impair* or ill* or sick* or ailment or malady or syndrome* or infection* or mortality or morbidity or death or dead or injur* or accident* or pain or incident*).tw.

16. exp public health/

17. 15 or 16

18. (quantitative or qualitative or empirical or data or statistic* or evidence or survey or stud* or interview or 'self report*' or poll* or experiment* or measure* or analyz* or analys* or 'focus group*' or question* or query or queri* or observation* or 'field stud*' or phenomenolog* or phenomenograph* or ethnolog* or ethnograph* or 'action research' or 'grounded theory' or 'case stud*' or 'multi?method*').mp.

19. exp qualitative research/ or exp quantitative analysis/ or exp empirical research/

20. exp health survey/

21. exp interview/ or exp semi structured interview/ or exp structured interview/ or exp telephone interview/ or exp unstructured interview/

22. exp self report/

23. exp questionnaire/ or exp open ended questionnaire/ or exp structured questionnaire/

24. methodology/ or exp case finding/ or exp grounded theory/ or exp multimethod study/ or exp quantitative study/ or exp sample size/

25. 18 or 19 or 20 or 21 or 22 or 23 or 24

26. (effect* or affect* or impact* or differ* or compliance or comply or adher* or implement* or influenc* or chang* or measure* or constrain* or screen* or control* or deter* or reduc* or increase* or decreas* or inflat* or vary or variation* or varie*).tw.

27. 4 and 14 and 17 and 25 and 26

**PsycINFO**

Total yield = 2105

1. celebrit*.ti,ab.

2. ((professional or elit* or famous or public or renown* or well-known or acclaim* or eminent or prominent or illustrious or recogniz* or reput* or influential or wealth* or power*) adj1 (person* or people or figure* or leader or athlete* or player or bodybuilder or sport* or basketball or football or hockey or baseball or soccer or Olympian or singer* or songwriter* or musician* or band or group or rapper* or artist* or actor* or actress or star or Hollywood or Bollywood or Nollywood or dancer or writer or author or comedian or performer or model* or supermodel* or chef or philanthropist or politic* or president or minister or king or queen or prince* or monarch)).ti,ab.

3. exp Celebrities/

4. 1 or 2 or 3

5. (communicat* or promot* or endors* or advert* or convinc* or market* or persua* or dissua* or sell* or sale* or publici* or awareness or campaign or "media coverage" or announc* or message* or disclos* or advoca* or advis* or advice or counsel* or educat* or instruct* or teach* or inform* or misinform* or prevent* or learn* or behavior?r* or act* or practice* or habit* or lifestyle* or regime or choice* or decision* or prefer* or attitude* or know* or belief* or perception* or view* or react* or response or respond* or stigma* or understand* or opinion* or litera* or illitera* or misunderstand* or misconcept* or misconstruct* or disbelief).tw.

6. exp health promotion/ or exp health attitudes/ or exp health behavior/ or exp health education/ or exp health knowledge/ or exp health literacy/ or exp lifestyle changes/ or exp preventive medicine/ or exp public service announcements/

7. exp information seeking/ or exp information literacy/

8. exp Public Opinion/

9. 5 or 6 or 7 or 8

10. (health or wellness or wellbeing or aging or longevity or disorder* or disease* or cancer or epidemic or pandemic or disability or impair* or ill* or sick* or ailment or malady or syndrome* or infection* or mortality or morbidity or death or dead or injur* or accident* or pain or incident*).tw.

11. exp public health services/ or exp public health/

12. 10 or 11

13. (quantitative or qualitative or empirical or data or statistic* or evidence or survey or stud* or interview or 'self report*' or poll* or experiment* or measure* or analyz* or analys* or 'focus group*' or question* or query or queri* or observation* or 'field stud*' or phenomenolog* or phenomenograph* or ethnolog* or ethnograph* or 'action research' or 'grounded theory' or 'case stud*' or 'multi?method*').mp.

14. exp surveys/ or exp mail surveys/ or exp telephone surveys/

15. exp interviewing/ or exp interviews/ or exp questioning/

16. exp empirical methods/ or exp experimental methods/ or exp observation methods/ or exp qualitative research/ or exp quantitative methods/

17. exp data collection/ or exp "sampling (experimental)"/ or exp statistical measurement/

18. exp ethnography/ or exp ethnology/

19. exp Phenomenology/

20. 13 or 14 or 15 or 16 or 17 or 18 or 19

21. (effect* or affect* or impact* or differ* or compliance or comply or adher* or implement* or influenc* or chang* or measure* or constrain* or screen* or control* or deter* or reduc* or increase* or decreas* or inflat* or vary or variation* or varie*).tw.

22. 4 and 9 and 12 and 20 and 21

**PubMed**

Total yield = 533

("famous persons"[MeSH Terms] OR celebrit*[Title/Abstract] OR ((professional OR elit* OR famous OR public OR renown* OR well-known OR acclaim* OR eminent OR prominent OR illustrious OR recogniz* OR reput* OR influential OR wealth* OR power*) AND (person* OR people OR figure* AND athlete OR leader OR athlete* OR player OR bodybuilder OR sport* OR basketball OR football OR hockey OR baseball OR soccer OR olympian OR singer* OR songwriter* OR musician* OR band OR group OR rapper* OR artist* OR actor* OR actress OR star OR hollywood OR hollywood OR hollywood OR dancer OR writer OR author OR comedian OR performer OR model* OR supermodel* OR chef OR philanthropist OR politic* OR president OR minister OR king OR queen OR prince* OR monarch) title/abstract))) AND (health OR wellness OR wellbeing OR aging OR longevity OR disorder* OR disease* OR cancer OR epidemic OR pandemic OR disability OR impair* OR ill* OR sick* OR ailment OR malady OR syndrome* OR infection* OR mortality OR morbidity OR death OR dead OR injur* OR accident* OR pain OR incident*) AND (communicat* OR promot* OR endors* OR advert* OR convinc* OR market* OR persua* OR dissua* OR sell* OR sale* OR publici* OR awareness OR campaign OR "media coverage" OR announc* OR message* OR disclos* OR advoca* OR advis* OR advice OR counsel* OR educat* OR instruct* OR teach* OR inform* OR misinform* OR prevent* OR learn* OR behavior?r* OR act* OR practice* OR habit* OR lifestyle* OR regime OR choice* OR decision* OR preference* OR attitude* OR know* OR belief* OR perception* OR view* OR react* OR respons* OR stigma* OR understand* OR opinion* OR litera* OR illitera* OR misunderstand* OR misconcept* OR misconstruct* OR disbelief OR "public health/prevention and control"[MeSH Terms] OR "public health/education"[MeSH Terms] OR "health communication"[MeSH Terms] OR "health education"[MeSH Terms] OR "health educators"[MeSH Terms] OR "health promotion"[MeSH Terms] OR "health literacy"[MeSH Terms] OR "information dissemination"[MeSH Terms] OR "information literacy"[MeSH Terms] OR "health knowledge, attitudes, practice"[MeSH Terms] OR "health behavior"[MeSH Terms]) AND (quantitative OR qualitative OR empirical OR data OR statistic* OR evidence OR stud* OR "multi?method*" OR survey* OR interview OR "self report*" OR poll* OR experiment* OR measure* OR analyz* OR analys* OR "focus group*" OR question* OR query OR queri* OR observation* OR "field stud*" OR phenomenolog* OR phenomenograph* OR ethnolog* OR ethnograph* OR "action research" OR "grounded theory" OR "case stud*" OR "empirical research"[MeSH Terms] OR "qualitative research"[MeSH Terms]) AND (effect* OR affect* OR impact* OR differ* OR compliance OR comply* OR adher* OR implement* OR influenc* OR chang* OR measure* OR constrain* OR screen* OR control* OR deter* OR reduc* OR increase* OR decreas* OR inflat* OR vary OR variation* OR varie*)

**CINAHL**

Total yield = 301

1. (MH "Public Figures")

2. AB ((professional or elit* or famous or public or renown* or well-known or acclaim* or eminent or prominent or illustrious or recogniz* or reput* or influential or wealth* or power*) adj1 (person* or people or figure* or leader or athlete* or player or bodybuilder or sport* or basketball or football or hockey or baseball or soccer or Olympian or singer* or songwriter* or musician* or band or group or rapper* or artist* or actor* or actress or star or Hollywood or Bollywood or Nollywood or dancer or writer or author or comedian or performer or model* or supermodel* or chef or philanthropist or politic* or president or minister or king or queen or prince* or monarch))

3. AB celebrit*

4. 1 OR 2 OR 3

5. (MH "Health Promotion+")

6. (MH "Health Education")

7. (MH "Health Knowledge") OR (MH "Health Knowledge and Behavior (Iowa NOC) (Non-Cinahl)+") OR (MH "Knowledge: Health Behaviors (Iowa NOC)")

8. (MH "Attitude to Health+") OR (MH "Attitude to Illness+")

9. (MH "Health Behavior+")

10. (MH "Preventive Health Care+")

11. TX (communicat* or promot* or endors* or advert* or convinc* or market* or persua* or dissua* or sell* or sale* or publici* or awareness or campaign or "media coverage" or announc* or message* or disclos* or advoca* or advis* or advice or counsel* or educat* or instruct* or teach* or inform* or misinform* or prevent* or learn* or behavior?r* or act* or practice* or habit* or lifestyle* or regime or choice* or decision* or prefer* or attitude* or know* or belief* or perception* or view* or react* or response or respond* or stigma* or understand* or opinion* or litera* or illitera* or misunderstand* or misconcept* or misconstruct* or disbelief)

12. 5 OR 6 OR 7 OR 8 OR 9 OR 10 OR 11

13. (MH "Public Health+")

14. TX (health or wellness or wellbeing or aging or longevity or disorder* or disease* or cancer or epidemic or pandemic or disability or impair* or ill* or sick* or ailment or malady or syndrome* or infection* or mortality or morbidity or death or dead or injur* or accident* or pain or incident*)

15. 13 OR 14

16. ""(quantitative or qualitative or empirical or data or statistic* or evidence or stud* or survey or interview or 'self report*' or poll* or experiment* or measur* or analyz* or analys* or 'focus group*' or question* or query or queri* or observation* or 'field stud*' or phenomenolog* or phenomenograph* or ethnolog* or ethnograph* or 'action research' or 'grounded theory' or 'case stud*' or 'multi method*')""

17. (MH "Quantitative Studies") OR (MH "Multimethod Studies") OR (MH "Models, Statistical") OR (MH "Meta Analysis")

18. (MH "Qualitative Studies+") OR (MH "Phenomenology")

19. (MH "Empirical Research")

20. (MH "Ethnographic Research")

21. (MH "Survey Research") OR (MH "Interviews") OR (MH "Structured Interview") OR (MH "Unstructured Interview") OR (MH "Semi-Structured Interview") OR (MH "Surveys")

22. (MH "Case Studies") OR (MH "Field Studies")

23. 16 OR 17 18 OR 19 OR 20 OR 21 OR 22

24. TX (effect* or affect* or impact* or differ* or compliance or comply or adher* or implement* or influenc* or chang* or measure* or constrain* or screen* or control* or deter* or reduc* or increase* or decreas* or inflat* or vary or variation* or varie*)

25. 4 AND 12 AND 15 AND 23 AND 24

Social Sciences Databases

Date of searches: 2014-07-31

**Communication Source**

Total yield = 499

AB ( (celebrit* or ((professional or elit* or famous or public or renown* or well-known or acclaim* or eminent or prominent or illustrious or recogniz* or reput* or influential or wealth* or power*) N1 (person* or people or figure* athlete or leader or athlete* or player or bodybuilder or sport* or basketball or football or hockey or baseball or soccer or Olympian or singer* or songwriter* or musician* or band or group or rapper* or artist* or actor* or actress or star or Hollywood or Bollywood or Nollywood or dancer or writer or author or comedian or performer or model* or supermodel* or chef or philanthropist or politic* or president or minister or king or queen or prince* or monarch))) ) AND TX ( (DE "PUBLIC interest" OR DE "PUBLIC opinion" or DE "HEALTH risk assessment -- Press coverage" OR DE "HEALTH risk communication" OR DE "MASS media & propaganda" OR DE "MASS media & public opinion" OR DE "MASS media & publicity" OR DE "MASS media & education" OR DE "MASS media & educators" OR DE "COMMUNICATION & education" OR DE "ENDORSEMENTS in advertising" OR DE "ADVERTISING campaigns" OR DE "ADVERTISING in educational media" OR DE "COMMUNICATION in medicine" OR DE "COMMUNICATION in health education" or communicat* or promot* or endors* or advert* or convinc* or market* or persua* or dissua* or sell* or sale* or publici* or awareness or campaign or ‘media coverage’ or announc* or message* or disclos* or advoca* or advis* or advice or counsel* or educat* or instruct* or teach* or inform* or misinform* or prevent* or learn* or behavio?r* or act* or practice* or habit* or lifestyle* or regime or choice* or decision* or prefer* or attitude* or know* or belief* or perception* or view* or react* or response* or respond or stigma* or understand* or opinion* or litera* or illitera* or misunderstand* or misconcept* or misconstruct* or disbelief) ) AND ( (DE "QUANTITATIVE research" OR DE "QUALITATIVE research" AND DE "INTERVIEWING" OR DE "QUESTIONING" OR DE "FOCUS groups" OR DE "QUESTIONNAIRES" OR DE "SURVEYS" OR DE "COMMUNICATION surveys" OR DE "SOCIAL surveys" OR DE "PUBLIC opinion polls" OR DE "PHENOMENOLOGY" or quantitative or qualitative or empirical or data or statistic* or evidence or stud* or “multi?method*” or survey* or interview or “self report*” or poll* or sampl* or experiment* or measure* or analyz* or analys* or 'focus group*' or question* or query or queri* or observation* or “field stud*” or phenomenolog* or phenomenograph* or ethnolog* or ethnograph* or “action research” or “grounded theory” or “case stud*”) ) AND ((effect* or affect* or impact* or differ* or compliance or comply or adher* or implement* or influenc* or chang* or measure* or constrain* or screen* or control* or deter* or reduc* or increase* or decreas* or inflat* or vary or variation* or varie*) ) AND ( (health or wellness or wellbeing or aging or longevity or disorder* or disease* or cancer or epidemic or pandemic or disability or impair* or ill* or sick* or ailment or malady or syndrome* or infection* or mortality or morbidity or death or dead or injur* or accident* or pain or incident*) )

**Sociological Abstracts**

Total yield = 3892

(((AB(professional) OR AB(elite*) OR AB(famous) OR AB(public) OR AB(renown*) OR AB(well-known) OR AB(acclaim*) OR AB(eminent) OR AB(prominent) OR AB(illustrious) OR AB(recognize*) OR AB(repute*) OR AB(influential) OR AB(wealth*) OR AB(power*)) NEAR/1 (AB(athlete*) OR AB(player) OR AB(bodybuilder) OR AB(sport*) OR AB(basketball) OR AB(football) OR AB(hockey) OR AB(baseball) OR AB(soccer) OR AB(Olympian) OR AB(singer*) OR AB(songwriter*) OR AB(musician*) OR AB(band) OR AB(group) OR AB(rapper*) OR AB(artist*) OR AB(actor*) OR AB(actress) OR AB(start*) OR AB(Hollywood) OR AB(Bollywood) OR AB(Nollywood) OR AB(dancer) OR AB(writer) OR AB(author) OR AB(comedian) OR AB(performer) OR AB(model*) OR AB(supermodel*) OR AB(chef) OR AB(philanthropist) OR AB(politic*) OR AB(president) OR AB(minister) OR AB(king) OR AB(queen) OR AB(prince*) OR AB(monarch))) OR SU.EXACT.EXPLODE("Fame") OR AB(celebrity*)) AND (SU.EXACT.EXPLODE("Public Health") OR all(health) OR all(wellness) OR all(wellbeing) OR all(aging) OR all(longevity) OR all(disorder*) OR all(disease*) OR all(cancer) OR all(epidemic) OR all(pandemic) OR all(disability) OR all(impair*) OR all(ill*) OR all(sick*) OR all(ailment) OR all(malady) OR all(syndrome*) OR all(infection*) OR all(mortality) OR all(morbidity) OR all(death) OR all(dead) OR all(injury*) OR all(accident*) OR all(pain) OR all(incident*)) AND (SU.EXACT.EXPLODE("Health Education") OR SU.EXACT.EXPLODE("Health Behavior" OR "Illness Behavior") OR all(communicat*) or all(promot*) or all(endors*) or all(advert*) or all(convinc*) or all(market*) or all(persua*) or all(dissua*) or all(sell*) or all(sale*) or all(publici*) or all(awareness) or all(campaign) or all(‘media coverage’) or all(announc*) or all(message*) or all(disclos*) or all(advoca*) or all(advis*) or all(advice) or all(counsel*) or all(educat*) or all(instruct*) or all(teach*) or all(inform*) or all(misinform*) or all(prevent*) or all(learn*) or all(behavio?r*) or all(act*) or all(practice*) or all(habit*) or all(lifestyle*) or all(regime) or all(choice*) or all(decision*) or all(prefer*) or all(attitude*) or all(know*) or all(belief*) or all(perception*) or all(view*) or all(react*) or all(response*) or all(respond) or all(stigma*) or all(understand*) or all(opinion*) or all(litera*) or all(illitera*) or all(misunderstand*) or all(misconcept*) or all(misconstruct*) or all(disbelief)) AND (SU.EXACT("Grounded Theory") OR SU.EXACT("Case Studies") OR SU.EXACT.EXPLODE("Qualitative Methods") OR SU.EXACT("Quantitative Methods") OR SU.EXACT("Sampling") OR SU.EXACT("Statistics") OR SU.EXACT("Measurement") OR SU.EXACT("Scientific Research") OR SU.EXACT("Empirical Methods") OR all(quantitative) or all(qualitative) or all(empirical) or all(data) or all(statistic*) or all(evidence) or all(stud*) or all(“multi?method*”) or all(survey*) or all(interview) or all(“self report*”) or all(poll*) or all(sampl*) or all(experiment*) or all(measure*) or all(analyz*) or all(analys*) or all('focus group*') or all(question*) or all(query) or all(queri*) or all(observation*) or all(“field stud*”) or all(phenomenolog*) or all(phenomenograph*) or all(ethnolog*) or all(ethnograph*) or all(“action research”) or all(“grounded theory”) or all(“case stud*”)) AND (all(effect*) or all(affect*) or all(impact*) or all(difference) or all(compliance) or all(comply) or all(adher*) or all(implement*) or all(influenc*) or all(chang*) or all(measure*) or all(constrain*) or all(screen*) or all(control*) or all(deter*) or all(reduc*) or all(increase*) or all(decreas*) or all(inflat*) or all(vary) or all(variation*) or all(varie*))

**Social Sciences Citation Index**

Total yield = 5884

((((**TOPIC:**(celebrit* OR (((((((((((((((professional OR elit*) OR famous) OR public) OR renown*) OR well-known) OR acclaim*) OR eminent) OR prominent) OR illustrious) OR recogniz*) OR reput*) OR influential) OR wealth*) OR power*) NEAR ((((((((((((((((((((((((((((((((((((((((((person* OR people) OR figure*) OR leader) OR athlete*) OR player) OR bodybuilder) OR sport*) OR basketball) OR football) OR hockey) OR baseball) OR soccer) OR Olympian) OR singer*) OR songwriter*) OR musician*) OR band) OR group) OR rapper*) OR artist*) OR actor*) OR actress) OR star) OR Hollywood) OR Bollywood) OR hollywood) OR dancer) OR writer) OR author) OR comedian) OR performer) OR model*) OR supermodel*) OR chef) OR philanthropist) OR politic*) OR president) OR minister) OR king) OR queen) OR prince*) OR monarch)))*AND* **TOPIC:** (((((((((((((((((((((((((health OR wellness) OR wellbeing) OR aging) OR longevity) OR disorder*) OR disease*) OR cancer) OR epidemic) OR pandemic) OR disability) OR impair*) OR ill*) OR sick*) OR ailment) OR malady) OR syndrome*) OR infection*) OR mortality) OR morbidity) OR death) OR dead) OR injur*) OR accident*) OR pain) OR incident*)) *AND* **TOPIC:**((((((((((((((((((((((((((((((((((((((((((((((((((((communicat* OR promot*) OR endors*) OR advert*) OR convinc*) OR market*) OR persua*) OR dissua*) OR sell*) OR sale*) OR publici*) OR awareness) OR campaign) OR "media coverage") OR announc*) OR message*) OR disclos*) OR advoca*) OR advis*) OR advice) OR counsel*) OR educat*) OR instruct*) OR teach*) OR inform*) OR misinform*) OR prevent*) OR learn*) OR behavior?r*) OR act*) OR practice*) OR habit*) OR lifestyle*) OR regime) OR choice*) OR decision*) OR preference*) OR attitude*) OR know*) OR belief*) OR perception*) OR view*) OR react*) OR respons*) OR stigma*) OR understand*) OR opinion*) OR litera*) OR illitera*) OR misunderstand*) OR misconcept*) OR misconstruct*) OR disbelief)) *AND* **TOPIC:**((((((((((((((((((((((((((((quantitative OR qualitative) OR empirical) OR data) OR statistic*) OR evidence) OR stud*) OR multimethod*) OR survey*) OR interview) OR "self report*") OR poll*) OR experiment*) OR measure*) OR analyz*) OR analys*) OR "focus group*") OR question*) OR query) OR queri*) OR observation*) OR "field stud*") OR phenomenolog*) OR phenomenograph*) OR ethnolog*) OR ethnograph*) OR "action research") OR "grounded theory") OR "case stud*")) *AND* **TOPIC:**(((((((((((((((((((((effect* OR affect*) OR impact*) OR difference*) OR compliance) OR comply) OR adher*) OR implement*) OR influenc*) OR chang*) OR measure*) OR constrain*) OR screen*) OR behavio?r*) OR deter*) OR reduc*) OR increase*) OR decreas*) OR inflat*) OR vary) OR variation*) OR varie*))

**Journals @ Scholars Portal**

Total yield = 421

(celebrit* OR "(professional OR elit* OR famous OR public OR renown* OR well-known OR acclaim* OR eminent OR prominent OR illustrious OR recogniz* OR reput* OR influential OR wealth* OR power*) N1 (person* OR people OR figure* OR leader OR athlete* OR player OR bodybuilder OR sport* OR basketball OR football OR hockey OR baseball OR soccer OR Olympian OR singer* OR songwriter* OR musician* OR band OR group OR rapper* OR artist* OR actor* OR actress OR star OR Hollywood OR Bollywood OR Nollywood OR dancer OR writer OR author OR comedian OR performer OR model* OR supermodel* OR chef OR philanthropist OR politic* OR president OR minister OR king OR queen OR prince* OR monarch)) AND (health OR wellness OR wellbeing OR aging OR longevity OR disorder* OR disease* OR cancer OR epidemic OR pandemic OR disability OR impair* OR ill* OR sick* OR ailment OR malady OR syndrome* OR infection* OR mortality OR morbidity OR death OR dead OR injur* OR accident* OR pain OR incident*) AND (communicat* OR promot* OR endors* OR advert* OR convinc* OR market* OR persua* OR dissua* OR sell* OR sale* OR publici* OR awareness OR campaign OR "media coverage" OR announc* OR message* OR disclos* OR advoca* OR advis* OR advice OR counsel* OR educat* OR instruct* OR teach* OR inform* OR misinform* OR prevent* OR learn* OR behavio?r* OR act* OR practice* OR habit* OR lifestyle* OR regime OR choice* OR decision* OR prefer* OR attitude* OR know* OR belief* OR perception* OR view* OR react* OR respons* OR stigma* OR understand* OR opinion* OR litera* OR illitera* OR misunderstand* OR misconcept* OR misconstruct* OR disbelief) AND (quantitative OR qualitative OR empirical OR data OR statistic* OR evidence OR stud* OR multi?method* OR survey* OR interview OR ‘self report*’ OR poll* OR experiment* OR measure* OR analyz* OR analys* OR 'focus group*' OR question* OR query OR queri* OR observation* OR 'field stud*' OR phenomenolog* OR phenomenograph* OR ethnolog* OR ethnograph* OR "action research" OR "grounded theory" OR "case stud*") AND (effect* OR affect* OR impact* OR difference* OR compliance OR comply* OR adher* OR implement* OR influenc* OR chang* OR measure* OR constrain* OR screen* OR control* OR deter* OR reduc* OR increase* OR decreas* OR inflat* OR vary OR variation* OR varie*)

Grey Literature

Date of search: 2014-07-31

**Proquest Dissertations & Theses A&I**

Yield = 680

ab(celebrity* OR ((professional OR elite* OR famous OR public OR renown* OR wellknown OR acclaim* OR eminent OR prominent OR illustrious OR recognize* OR repute* OR influential OR wealth* OR power*) N1 (person* OR people OR figure* athlete OR leader OR athlete* OR player OR bodybuilder OR sport* OR basketball OR football OR hockey OR baseball OR soccer OR Olympian OR singer* OR songwriter* OR musician* OR band OR group OR rapper* OR artist* OR actor* OR actress OR start OR Hollywood OR Bollywood OR Nollywood OR dancer OR writer OR author OR comedian OR performer OR model* OR supermodel* OR chef OR philanthropist OR politic* OR president OR minister OR king OR queen OR prince* OR monarch))) AND ft(communicat* OR promot* OR endors* OR advert* OR convinc* OR market* OR persua* OR dissua* OR sell* OR sale* OR publici* OR awareness OR campaign OR "media coverage" OR announc* OR message* OR disclos* OR advoca* OR advis* OR advice OR counsel* OR educat* OR instruct* OR teach* OR inform* OR misinform* OR prevent* OR learn* OR behavio?r* OR act* OR practice* OR habit* OR lifestyle* OR regime OR choice* OR decision* OR prefer* OR attitude* OR know* OR belief* OR perception* OR view* OR react* OR respons* OR stigma* OR understand* OR opinion* OR litera* OR illitera* OR misunderstand* OR misconcept* OR misconstruct* OR disbelief) AND ft(health OR wellness OR wellbeing OR aging OR longevity OR disorder* OR disease* OR cancer OR epidemic OR pandemic OR disability OR impair* OR ill* OR sick* OR ailment OR malady OR syndrome* OR infection* OR mortality OR morbidity OR death OR dead OR injur* OR accident* OR pain OR incident*) AND ft(quantitative OR qualitative OR empirical OR data OR statistic* OR evidence OR stud* OR multi?method* OR survey* OR interview OR ‘self report*’ OR poll* OR experiment* OR measure* OR analyz* OR analys* OR 'focus group*' OR question* OR query OR queri* OR observation* OR 'field stud*' OR phenomenolog* OR phenomenograph* OR ethnolog* OR ethnograph* OR "action research" OR "grounded theory" OR "case stud*") AND ft(effect* OR affect* OR impact* OR difference* OR compliance OR comply* OR adher* OR implement* OR influenc* OR chang* OR measure* OR constrain* OR screen* OR control* OR deter* OR reduc* OR increase* OR decreas* OR inflat* OR vary OR variation* OR varie*)
