# Supplementary material for: Celebrities’ impact on health-related knowledge, attitudes, behaviors, and status outcomes: protocol for a systematic review, meta-analysis, and meta-regression analysis
Source: Syst Rev. 2017 Jan 21;6:13. doi: 10.1186/s13643-016-0395-1 (PMC5251292; doi:10.1186/s13643-016-0395-1)
Supplement: Additional file 4: — Title and abstract screening form. (DOCX 80 kb) [file 13643_2016_395_MOESM4_ESM.docx]

**Additional File 4**

Title and Abstract Screening Form

**Screener Initials __________**

**General Information**

| Title: |  |
| --- | --- |
| First author (or group label) |  |
| Journal name: |  |
| Year: |  |
| Volume: |  |
| Issue: |  |
| Pages: |  |
| Reference Manager Citation Number: |  |

**Eligibility**

| Does the study explore a potential association with celebrities as the intervention (independent variable)? | **Yes** _______  **No** _______ |
| --- | --- |
| Does the study explore a potential association with a health-related knowledge, attitude, or behaviour as the outcome (dependent variable)? (Note that health-related behaviours encompass a wide range of actions that can either promote or threaten one's health) | **Yes** _______  **No** _______ |
| Does this study employ empirical methods? Indicate YES if study employs either quantitative or qualitative methods. Indicate NO if there is no empirical data gathered (ex: a narrative review). | **Yes** _______  **No** _______ |

Eligible for data abstraction if, and only if, the answer is **YES** to all three questions. Review any discrepancies on eligibility that cannot be resolved through discussion with the project arbitrator (Steven Hoffman).

**PAPER IS ELIGIBLE FOR FULL TEXT SCREENING: Yes** _______  **No** _______
