# Supplementary material for: Celebrities’ impact on health-related knowledge, attitudes, behaviors, and status outcomes: protocol for a systematic review, meta-analysis, and meta-regression analysis
Source: Syst Rev. 2017 Jan 21;6:13. doi: 10.1186/s13643-016-0395-1 (PMC5251292; doi:10.1186/s13643-016-0395-1)
Supplement: Additional file 5: — Full-text screening form. (DOCX 81 kb) [file 13643_2016_395_MOESM5_ESM.docx]

**Additional File 5**

Full Text Screening Form

**Screener Initials __________**

**General Information**

| Title: |  |
| --- | --- |
| First author (or group label) |  |
| Journal name: |  |
| Year: |  |
| Volume: |  |
| Issue: |  |
| Pages: |  |
| Reference Manager Citation Number: |  |

**Eligibility**

| Does the study explore an association with celebrities as the intervention (independent variable)? | **Yes** _______  **No** _______ |
| --- | --- |
| Does the study explore an association with a health knowledge, attitude, or health-related behaviour as the outcome (dependent variable)? (Note that health-related behaviors encompass a wide range of actions that can either promote or threaten one's health) | **Yes** _______  **No** _______ |
| Is this a peer-reviewed primary study that employs empirical methods? Indicate YES if study employs either quantitative or qualitative methods to gather original data. Indicate NO if there is no empirical data gathered (ex: a narrative review). | **Yes** _______  **No** _______ |

Eligible for data abstraction if, and only if, the answer is **YES** to all three questions. Review any discrepancies on eligibility that cannot be resolved through discussion with the project arbitrator (Steven Hoffman).

**PAPER IS ELIGIBLE FOR DATA ABSTRACTION: Yes** _______  **No** _______
