# Supplementary material for: Celebrities’ impact on health-related knowledge, attitudes, behaviors, and status outcomes: protocol for a systematic review, meta-analysis, and meta-regression analysis
Source: Syst Rev. 2017 Jan 21;6:13. doi: 10.1186/s13643-016-0395-1 (PMC5251292; doi:10.1186/s13643-016-0395-1)
Supplement: Additional file 6: — Data abstraction form —phase 1. The first data abstraction form that will be utilized with all studies in order to collect population, intervention, methodology, and preliminary outcome information. (DOCX 111 kb) [file 13643_2016_395_MOESM6_ESM.docx]

**Additional File 6**

Data Abstraction Form – Phase 1

**General Information**

| **Title:** |  |
| --- | --- |
| **First author (or group label)** |  |
| **Journal name:** |  |
| **Year:** |  |
| **Volume:** |  |
| **Issue:** |  |
| **Pages:** |  |
| **Reference Manager Citation Number:** |  |

**Section 1: Demographics**

| **1. How many participants were in this study?**  Note: indicate only the number of participants analyzed/included after drop-outs and non-responding |  |
| --- | --- |
| **2. What is the mean age of the study participants?** |  |
| **3. What is the standard deviation on the mean age of the study participants?** |  |
| **4. What is the age range of the study participants?** |  |
| **5. What % of the study participants were male?** |  |
| **6. Were demographic data described separately based on groupings? (e.g. by intervention group, by sex)** | Yes  No  Can’t tell (Specify: ____) |
| **7. If Y to 6, use this space to identify the demographic data for each group.** Note: Report the groups in the order in which they appear in the text. | Name of group 1:  # of participants in group 1:  Mean age and SD group 1:  Age range in group 1:  % Male in group 1:  (repeat for further groups) |
| **8. Did the study participants all belong to the same race/ethnicity?** | Yes  No  Can’t tell (Specify: ____) |
| **9. If Y to 8, which race/ethnicity did the study participants belong to?** |  |
| **10. Did the study participants all share a particular health-related characteristic (disease state, condition, etc.)?** | Yes  No  Can’t tell (Specify: ____) |
| **11. If Y to 10, which health-related characteristic was shared by all of the participants?** |  |
| **12. Are there any other descriptors of the study population?** | Yes  No  Can’t tell (Specify: ____) |
| **13. If Y to 12, list the name of the variables on which additional descriptors of the population were described. Do not report the actual proportion of people that belong to each category in the variables.**  Eg) education; socioeconomic status; employment status |  |
| **13. List which criteria was used to INCLUDE participants in the study.**  Format: **criteria 1; criteria 2; criteria 3**  Eg) Provided consent; Attended university |  |
| **14. List which criteria was used to EXCLUDE participants from the study.**  Format: **Criteria 1; Criteria 2; Criteria 3**  Eg) Not english-speaking; less than 10 years of age |  |

**Section 2: Setting**

| **15. When did this study begin their data collection?**  Note: if DD/MM not applicable then just specify YYYY or MM/YY | DD/MM/YYYY |
| --- | --- |
| \| **16. When did this study end their data collection?**  Note: if DD/MM not applicable then just specify YYYY or MM/YY \| \| --- \| | DD/MM/YYYY |
| **17. In which country did this study take place?**  Note: If multiple countries, separate each location by a semicolon.  Eg) Canada; USA |  |
| **18. What was the setting of the study?**  Examples of settings: elementary school, HIV/AIDS clinic, etc.  Note: If multiple settings, separate each setting by a semicolon.  Note 2: we will consider "Online" to be an appropriate setting |  |

**Section 3: Intervention**

| \| **19. Describe the celebrity intervention with as much detail as provided in the study. Be sure to specify where the celebrity involvement takes place.**  Note: For multicomponent interventions (where the celebrity was only a component of the full intervention) it is important here to specify what the other components of the intervention were as well.  Note 2: For studies that do not have an explicit intervention, state "No explicit intervention". Eg) A study may be measuring general attitudes about celebrity influence without referring to a specific celebrity-related event that had happened. \| \| --- \| |  |
| --- | --- | --- |
| **20. What category is this intervention? (Select as many as apply)**  Examples of categorization:   - Celebrity-led: celebrity disclosure of disease (note: suicide is NOT a celebrity-led intervention) - Media-led: news coverage of a suicide - Commercially-led: advertisements featuring celebrities - Partnership with an educational or public health body: educational TV shows, public service announcements from the government | Celebrity-led  Media-led  Commercial-led  Partnership with educational or public health body  No explicit intervention  Can’t tell (Specify: ________) |
| **21 If a specific celebrity was mentioned, what is the celebrity's first and last name?**  Note: If there are multiple celebrities, indicate the celebrity names in the order in which they appear in the text. Copy and paste names from the article wherever possible to avoid spelling errors.  Note 2: Type in **"Name unspecified"** if a celebrity was not named, but was still described (E.g. a study looking at the copycat suicide after a famous unnamed movie actor's death)  Note 3: If a group of celebrities was mentioned collectively, but no individual was mentioned, you can just write in "No individual celebrity: (Group name)" and then indicate the category they fall under in the next question.  Note 4: Indicate "No explicit intervention" when no particular celebrities were mentioned, and instead the study was about "celebrities" in general. |  |
| **22. For each celebrity mentioned in 21, indicate the celebrity type. Select as many as apply.** | Entertainment star  Politician  Religious leader  Athlete  Fashion model  Other (specify: _______) |
| **23. Was celebrity influence measured as an independent variable?**  Examples of measuring celebrity influence: Level of identification with a celebrity, amount of celebrity-related media consumption, celebrity worship  Note: general amount of media consumption will not be considered as a measurement of celebrity influence | Yes  No  Can’t tell (Specify; ____) |
| \| **24. If Y to 23, how was celebrity influence measured?**  Note: Do not describe the individual questions or the scoring system of the scale here.  Format: Name of the scale/measure used. Type of scale (e.g. survey, questionnaire, tool). Brief description of what the scale measures.  *Ex: Celebrity Attitude Scale. Survey. Identifies people's views of famous persons and quantifies celebrity worship.* \| \| --- \| |  |
| **25. If Y to 23, describe how to interpret the units on the scale (mentioned in 24) on which celebrity influence was measured.**  Format for surveys/scales: **# of domains/subscales on the survey and each of their names (if applicable), followed by the # of items in each domain/subscale. Total # of items.** **How responses for each item are recorded (Eg. Likert scale ranging from 1 to 5 where 1=strongly disagree and 5=strongly agree, yes/no, circling a number between 0 and 100 in intervals of 10 to show what percentage of the time this happens to them.** **Range of total scores, and how the total score is interpreted.**  Example of survey description (for celebrity attitude scale): 3 domains: Entertainment-social (7 items), Intense-personal (13 items), borderline-pathological (7 items). 34 items total. Responses to each item are in the format of a Likert scale ranging from 1 to 5, where 1=strongly disagree and 5= strongly agree. Total scores range from 1 to 170. <35 is normal, 35-100 is moderate pathological, >100 is pathological. |  |
| **26. Was the celebrity intervention communicated in a broad or targeted way?**  Eg) If the celebrity intervention was created specifically for the study or for a specific group of people, it is a targeted intervention.  Eg2) If the celebrity intervention was something that is widely accessible, like a billboard advertisement, it is a broad intervention | Broad  Targeted  Can’t tell (Specify:_____)  No explicit intervention |
| **27. How was the celebrity intervention communicated? (Select as many as are applicable)** | Online (social media, social networks, websites)  Print (newspapers, magazines, posters, product packaging, etc.)  Radio  Audiovisual (TV, movies, theatre)  Other (Specify:_____)  No explicit intervention  Can’t tell (Specify:______) |

**Methodology**

| \| \| **28. Was the nature of the study qualitative or quantitative?** \| \| --- \| \| \| --- \| --- \| | Quantitative  Qualitative  Both  Can’t Tell (Specify: _____) |
| --- | --- | --- | --- |
| **29. What was the study design?**  Refer to the Cochrane classification of study designs for definitions. | Cross-sectional  Randomized controlled trial  Quasi-randomized controlled trial  Controlled trial (2 group)  Controlled trial (1 group)  Time series  Case-control  Prospective cohort (2 group)  Prospective cohort (1 group)  Retrospective cohort (2 group)  Retrospective cohort (1 group)  Other (Specify: _______)  Can’t tell (Specify: _______) |
| **30. Was there a control group?**  Note: The control group must be a comparison group with no celebrity involvement.  Note 2: Outcome data must be provided for the control group in the same, complete way as it is provided for the intervention group.  Note 3: Data collected at a time point before a celebrity intervention is applied can be considered as a control group |  |
| **31. If Y to 30, describe the control group(s) with as much information possible.** |  |
| \| **32. Did the study carry out subgroup analysis?**  Note: A subgroup is a “cluster” of people (sharing a particular set of characteristics) that is analyzed from the larger intervention and/or control group. Clusters should be based on pre-defined variables.  Note 2: Study outcomes should be reported for each subgroup as they are reported for the intervention and control groups as a whole, as otherwise the subgroup data will not be of use to our review.  Note 3: Look carefully into whether all subgroup data was reported (e.g. in a table) and separated for each intervention group. Also, if one odds ratio is reported for each subgroup, that is fine, since OR is inherently comparing 2 groups. Be extra careful with this question. \| \| --- \| |  |
| **33. If Y to 32, name the subgroup(s) on which the data was analyzed (if multiple subgroups, separate them with a semicolon).**  Format: Subgroup 1 (Category1/Category2/Category3); Subgroup 2 (Category1/Category2/Category3)  Eg) Sex (Male/Female); Age(continuous); Race (White/Black/Hispanic/Other) |  |
| **34. How was data collected?** | Records  Survey/self-report  Both records and surveys/self-report  Can’t tell (Specify: _____)  Not Reported |
| **35. Was data collected continuously or at discrete time points?** | Continuously  Discrete  Can’t tell (Specify: _____)  Not Reported |
| **36. If answered “continuously” to 35, report how long before (if applicable, eg. for time series studies) and/or after the intervention was data collected continuously for.**  Note: Report only the time periods for which data is made available in the study. | Before the intervention:  After the intervention: |
| **37. If answered “discrete” to 35, list the time point(s) before/after the intervention at which the data was collected**  Note: Write N.R.  if not reported.  Note 2: If the specific time point is not mentioned, you can write the time point relative to the intervention and "time point unspecified" in brackets. Eg) *Before intervention (time point unspecified); After intervention (time point unspecified)*  Note 3: If data only collected once, please specify this as well (just fill out one box) | Time point 1:  Time point 2:  Time point 3:  Time point 4:  Time point 5:  Time point 6:  Time point 7:  Time point 8: |
| **38. What was the health-related issue addressed by this study (select as many as apply)**  Note: Try to be as liberal as possible when classifying themes. | Health conditions  Medical services and products  Substance use  Lifestyle and health decisions  Can’t tell (Specify: _____) |

**Additional Comments**

| **39. Do you have any additional comments or notes about this study?**  Eg) Data that you think should be included, special notes about this study, any confusion you may have had in using this form with this study |  |
| --- | --- |

All questions are mandatory. If it is not possible to answer a question, indicate the following:

N/A for items that are not applicable

N.R. for items that are not reported

D.V. if the variable is a dependent variable of the study
